# Effects of Protein Restriction and Succedent Realimentation on Jejunal Function and Bacterial Composition of Different Colonic Niches in Weaned Piglets

**DOI:** 10.3389/fvets.2022.877130

**Published:** 2022-05-03

**Authors:** Jue Wang, Yizhi Zhu, Shiyi Tian, Qing Shi, Huairong Yang, Jing Wang, Weiyun Zhu

**Affiliations:** ^1^Laboratory of Gastrointestinal Microbiology, National Center for International Research on Animal Gut Nutrition, National Experimental Teaching Demonstration Center of Animal Science, College of Animal Science and Technology, Nanjing Agricultural University, Nanjing, China; ^2^Laboratory of Stem Cells and Translational Medicine, School of Medicine, Institutes for Life Sciences, South China University of Technology, Guangzhou, China

**Keywords:** dietary protein, jejunal function, colonic microbiota, compensatory growth, piglets

## Abstract

Recent studies have proved that protein succedent realimentation could rescue the loss of growth performance in weaning piglets caused by a prior protein restriction. However, how the protein restriction and succedent realimentation influence the jejunal function and bacterial composition of different colonic niches microbiota in weaning piglets needs a further investigation. After protein succedent realimentation, we found that the treatment group (TRE) piglets had a higher IGF-1 content and *IGF-1R* gene expression level in jejunal mucosa than the control group (CON) piglets. The *ZO-1* gene expression level was up-regulated in the jejunal mucosa of TRE piglets during protein restriction and succedent realimentation, while the jejunal permeability of TRE piglets was only decreased after protein succedent realimentation. In addition, we found that protein restriction and succedent realimentation increased the gene expression of *Pept-1* and the fecal apparent digestibility of crude protein in TRE piglets, but decreased the fecal nitrogen content. After 16S rRNA MiSeq sequencing of bacteria in different colonic niches (mucosa and digesta), TRE piglets had a higher relative abundance of beneficial bacteria and a lower relative abundance of potential pathogens than CON piglets in different colonic niches after protein restriction and succedent realimentation. Our data showed that protein restriction and succedent realimentation decreased the concentrations of branch chain fatty acids and ammonia-N in the colon of TRE piglets. In addition, protein succedent realimentation increased the concentration of total short chain fatty acids in the colon of TRE piglets. All these findings demonstrated that the strategy of protein restriction and succedent realimentation is an effective way to improve intestinal health of weaning piglets, and provided new insights into the nutrition management of piglets during the weaning period.

## Introduction

Piglets experience an abruptly and earlier weaning process in the modern pig industry compared to those under natural growing conditions (1). During their weaning period, diet-shifting is an important factor that puts the piglets under dramatic stress, resulting in their gut dysfunction, bacterial infectious diarrhea, and growth retardation (2). Applying nutrition management to reduce the economic losses of weaning stress have always attracted attention for commercial swine production (3). It is commonly believed that maintaining the normal level of dietary protein is necessary for the growth of weaning piglets (4). However, due to insufficient nitrogen nutrient digestibility in the small intestine of weaning piglets, a lot of undigested dietary protein gets into the hind gut and is fermented by bacteria, thereby producing toxic metabolites including ammonia, indole, and various kinds of phenols (5, 6). These harmful fermentation by-products aggravate the weaning stresses of piglets by reducing intestinal epithelial integrity and causing host inflammation (6). In addition, those metabolites would also elevate fecal nitrogen levels and pollute the environment (7). To minimize the adverse effects of this problem, several studies applied a low-protein diet technology (or protein restriction) to relieve the weaning stress (8, 9). Although positive progress associated with maintaining enteric health has been achieved, the process resulted in the reduction of piglet growth. Is there a way to obtain the benefits of protein restriction without inhibiting the growth of weaning piglets?

Recent studies by Hou et al. and our group revealed that protein succedent realimentation was an effective way to rescue the loss of growth performance caused by the prior protein restriction (4, 10). These results demonstrated that protein restriction and succedent realimentation improved feed conversion, protein deposition, and intestinal health in weaning piglets (4, 10). Our group revealed that weaning piglets achieve compensatory growth after protein restriction and succedent realimentation through a mechanism closely related to hepatic growth hormone (GH)/insulin-like growth factors (IGF-1) signaling axis activation (11). The jejunum is the main site for digestion and absorption of dietary protein. However, how the protein restriction and succedent realimentation influenced the jejunal function and nitrogen nutrient digestibility in weaning piglets is still unclear. Previous studies also highlighted that colonic bacteria can modulate feeding behavior, hormone secretion, and host health through their metabolites (12, 13). Notably, diet-shifting would alter the composition of colonic bacteria (14). Thus, it is essential to investigate how the colonic bacterial composition and metabolism respond to the protein restriction and succedent realimentation in weaning piglets.

Here, we explored the effect of protein restriction and succedent realimentation on the jejunal function and nitrogen nutrient digestibility in the weaning piglets. To comprehensively reveal how the protein restriction and succedent realimentation affect the colonic bacterial composition and metabolites, we investigated the alteration of bacterial abundance in different colonic niches (mucosa and diegesta). In addition, the colonic lumenal concentrations of short chain fatty acids and ammonia-N were also measured in this study.

## Materials and Methods

The care procedures of all animals in our study were fulfilled by following the Experimental Animal Care and Use Guidelines of China, and *in vivo* experimental protocols were approved by the Animal Care and Use Committee of Nanjing Agricultural University (Nanjing, Jiangsu province, China).

### Animal Experimental Design and Sampling

A total of 36 piglets (Duroc × Landrace × Yorkshire) of both sexes (half castrated males and half females) were weaned at either 4 weeks of age and weighed 6.47 ± 0.04 kg, were used in the experiment. Then, according to their sex and body weight, they were randomly divided into a control group (CON) and a treatment group (TRE) with six pens per group and three piglets housed in each pen. The whole experiment lasted for 28 days and was divided into the protein restriction period (day 0–day 14) and the protein realimentation period (day 15–day 28). TRE piglets were fed a low protein level diet (13.05% crude protein) during the protein restriction period, and the diet protein content returned to the normal level (18.83% crude protein) during the protein realimentation period. Meanwhile, the CON piglets were fed a normal protein level diet for the whole experimental period. All piglets were raised under the same condition (free access to food and water) during the experimental period on a commercial pig farm in Jiangsu Province, China. The ingredient and nutrient composition of both diets are shown in Supplementary Table 1.

On days 9, 11, 13, 19, 21, and 23, fresh fecal samples of each group of piglets were collected and mixed in a sterile plastic bag, then 10 ml HCl (10%) solution was added to 100 g of feces to prevent the ammonia nitrogen evaporation. Fecal samples from days 9, 11, and 13 of each group were mixed to represent the protein restriction period, and fecal samples from days 19, 21, and 23 of each group were mixed to represent the protein realimentation period. The mixture was then kept at 20°C for the digestibility test. On day 14 and day 28, six piglets based on parameters like sex and average body weight were randomly selected from each group (one piglet per pen) for sampling. Blood samples were collected via jugular venipuncture using vacuum tubes and then centrifuged at 1,000 × *g* for 15 min to obtain serum. After the piglet was sacrificed, the jejunum and colon were ligated, separated, and removed. Digesta samples of the proximal colon were collected. The jejunum and colon tissue were excised and rinsed in ice-cold phosphate buffer saline (PBS), and their mucosal samples were scraped and collected. All samples were frozen in liquid nitrogen immediately and kept at −80°C until analysis.

### Chemical Analyses

As Yu et al. described, all feed and fecal samples were measured for crude protein (method 990.03) according to the procedure described by the Association of Official Analytical Chemists (AOAC) (15, 16). The total tract apparent digestibility (TTAD) was measured by using an acid-insoluble ash (AIA) as the internal standard in the diet and feces. The concentrations of AIA in the diets and feces were analyzed according to the procedure of the International Organization for Standards [method no.5985; (17) (ISO, 2003)]. The TTAD was calculated via the following formula: TTAD (%) = 100 - (A1 × F2)/(A2 × F1) × 100, where A1: the AIA content of the feed, A2: the AIA content of feces, F1: the nutrient content of the feed, and F2: the nutrient content of feces.

### DNA Extraction, 16S rRNA Gene Amplification, High-Throughput Sequencing, and Bioinformatics Analysis

The method of DNA extraction, 16 rRNA gene amplification, and high-throughput sequencing were the same as was employed in our previous study (18). Total genomic DNA of the colonic mucosa and colonic digesta was extracted by using the PowerSoil DNA Isolation kit (MoBio Laboratories, Carlsbad, U.S.), and DNA samples were stored at −80°C until further 16S rRNA MiSeq sequencing. A universal primer (F 5'-ACTCCTRCGGGAGGCAGCAG-3' and R 5'-GGACTACCVGGGTATCTAAT-3') was used to amplify the V3–V4 region of the bacterial 16S rRNA gene. The PCR process included an initial denaturation (95°C for 2 min), 25 cycles of denaturation (95°C for 30 s), annealing (55°C for 30 s), elongation (72°C for 30 s), and a final extension (72°C for 5 min). Then, the PCR products were purified by using an AxyPrep DNA Gel Extraction Kit (Axygen Biosciences, Union City, U.S.). Purified products were pooled in equimolar and paired-end sequences (2 × 250) on an Illumina MiSeq platform according to the standard protocols. As Wang's study described, Raw FASTQ files were de-multiplexed and quality-filtered using QIIME (version 1.70) with standard criteria (19). OTUs were clustered with a 97 % similarity cut-off using UPARSE (version 7.1 http://drive5.com/uparse/), and chimeric sequences were identified and removed using UCHIME.20. The most abundant sequences within each OTU were designated as representative sequences and were classified using the Ribosomal Database Project (RDP) classifier with a standard minimum support threshold of 80%. The diversity of colonic mucosal and digesta bacteria (indexes of Shannon and Simpson) was assessed using MOTHUR v.1.29.0.

### Measurements of Microbial Metabolites

As our previous report described, a gas chromatography method (Shimadzu, GC-14A with an FID detector, Japan) was used to determine the concentrations of short-chain fatty acids (SCFAs) in the colonic digesta (20). The ammonia-N concentration of colonic digesta in the weaning piglets was measured by the colorimetric method which was described by Nyachoti et al. (21).

### RNA Extraction and Quantitative Real-Time PCR

Total RNA of samples was extracted from colonic mucosa with TRIzol reagent (Takara Bio, Otsu, Japan) according to the manufacturer's instructions, then they were reverse-transcribed to complementary DNA (cDNA) using a PrimeScript RT reagent Kit (Takara Biotechnology Co., Ltd. Otsu, Japan) according to the recommended procedures. The expression of target genes was measured by quantitative real-time PCR with SYBR Premix Ex TagTM (Tli RnaseH Plus) qPCR kit (Takara Biotechnology Co., Ltd., Otsu, Japan) according to the manufacturer's guidelines, and the fluorescence was detected by a sequence detector system (ABI 7300 SDS; Foster City, U.S.) according to the description of a previous study (10). Primers in this study were synthesized from Invitrogen Life Technologies (Shanghai, China), and their sequences are shown in Supplementary Table 2. The 2^−ΔΔCt^ method was used to calculate the expression of target genes relative to the housekeeping gene (β-actin) (22).

### ELISA and Enzyme Activity

The concentrations of the growth hormone (GH), insulin, insulin-like growth factor-1 (IGF-1), and D-lactate in the different samples were determined by using ELISA kits (Angle Gene Biotechnology Co., Ltd., Nanjing, China) according to the manufacturer's instructions. The enzyme activities of the aminopeptidases A (APA), aminopeptidases N (APN), dipeptidyl peptidase-4 (DPP-4), and diamine oxidase (DAO) in different samples were measured by using the enzyme activity kits (Angle Gene Biotechnology Co., Ltd., Nanjing, China). For standardization, the concentrations of protein were determined by the BCA method using a protein assay kit (Nanjing Jiancheng Bioengineering Institute, Nanjing, China).

### Statistical Analysis

Data were analyzed with SPSS 25.0 and reported as mean ± SEM. The Student's *t*-test and non-parametric methods (Kruskal–Wallis test and Mann–Whitney *U*-test) were applied to compare the differences between the two groups. The normality of variable distribution was tested with the Shapiro–Wilk test. The variables that had a non-normal distribution were analyzed using the non-parametric methods. The *p-*value of the bacterial analysis was adjusted with false-discovery-rate (FDR) correction by the Benjamini-Hochberg method. A *p* < 0.05 was regarded as statistically significant.

## Results

### Effect of Protein Restriction and Succedent Realimentation on Hormone Contents and *IGF-1* Receptor Gene Expression of Jejunum in Weaned Piglets

**Figure 1:**
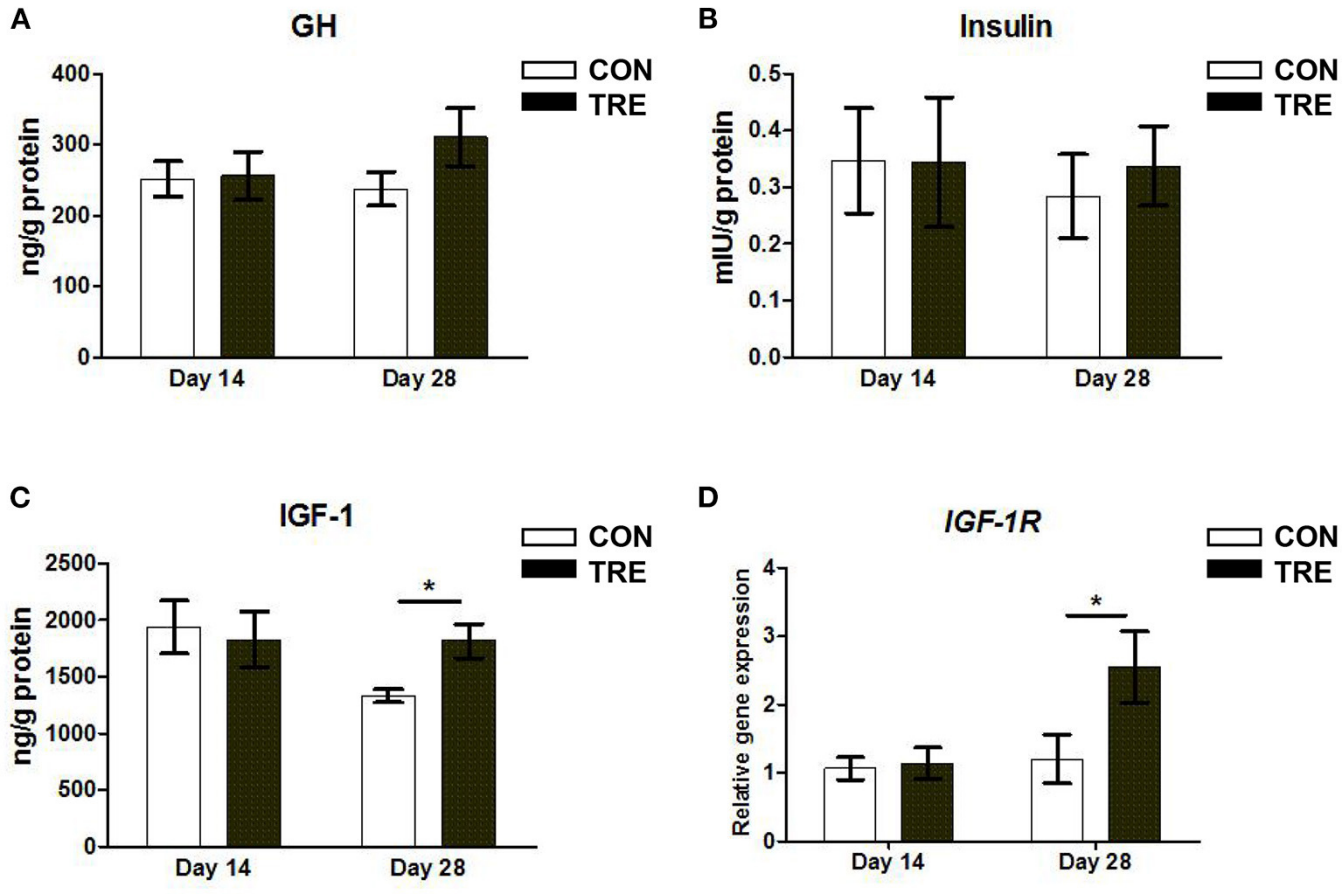
Effect of protein restriction and succedent realimentation on hormone contents and insulin-like growth factor-1 receptor gene expression of jejunum in weaned piglets (*n* = 6). **(A)** Concentration of growth hormone (GH) in jejunal mucosa on day 14 and 28. **(B)** Concentration of insulin in jejunal mucosa on day 14 and 28. **(C)** Concentration of insulin-like growth factor-1 (IGF-1) in jejunal mucosa on day 14 and 28. **(D)** Alterations of *IGF-1 receptor* (*IGF-1R*) gene expression in jejunal mucosa on day 14 and 28. *p* < 0.05 were considered significant and markers (*) between groups on the same day. CON, control group; TRE, treatment group.

Our previous studies demonstrated that protein restriction and succedent realimentation altered the GH-IGF1 signaling axis in rats and weaned piglets (11, 23). Hence, we first measured the concentration of GH, IGF-1, and insulin in jejunal mucosa. As shown in Figures 1A,B, neither protein restriction nor succedent realimentation affected the jejunal mucosal GH and insulin concentrations on day 14 and 28 (*p* > 0.05). In addition, the protein restriction did not significantly influence the alteration of IGF-1 concentration between the CON and TRE groups on day 14 (*p* > 0.05), while the protein succedent realimentation increased the IGF-1 concentration of the jejunal mucosa in TRE piglets on day 28 (*p* < 0.05; Figure 1C). Since IGF-1 receptors (IGF-1R) mediated IGF-1 biological activity in many tissues, we detected the gene expression level of *IGF-1R* in jejunal mucosa. As shown in Figure 1D, the protein restriction did not affect the alteration of *IGF-1R* gene expression level in the CON and TRE groups on day 14 (*p* > 0.05), but the *IGF-1* gene expression level of the jejunal mucosa in TRE piglets was significantly higher than that of CON piglets after protein succedent realimentation on day 28 (*p* < 0.05). These results indicated that TRE piglets had a higher IGF-1 concentration and *IGF-1R* gene expression level in jejunal mucosa than CON piglets during compensatory growth.

### Effect of Protein Restriction and Succedent Realimentation on the Barrier Function of Jejunum in Weaned Piglets

**Figure 2:**
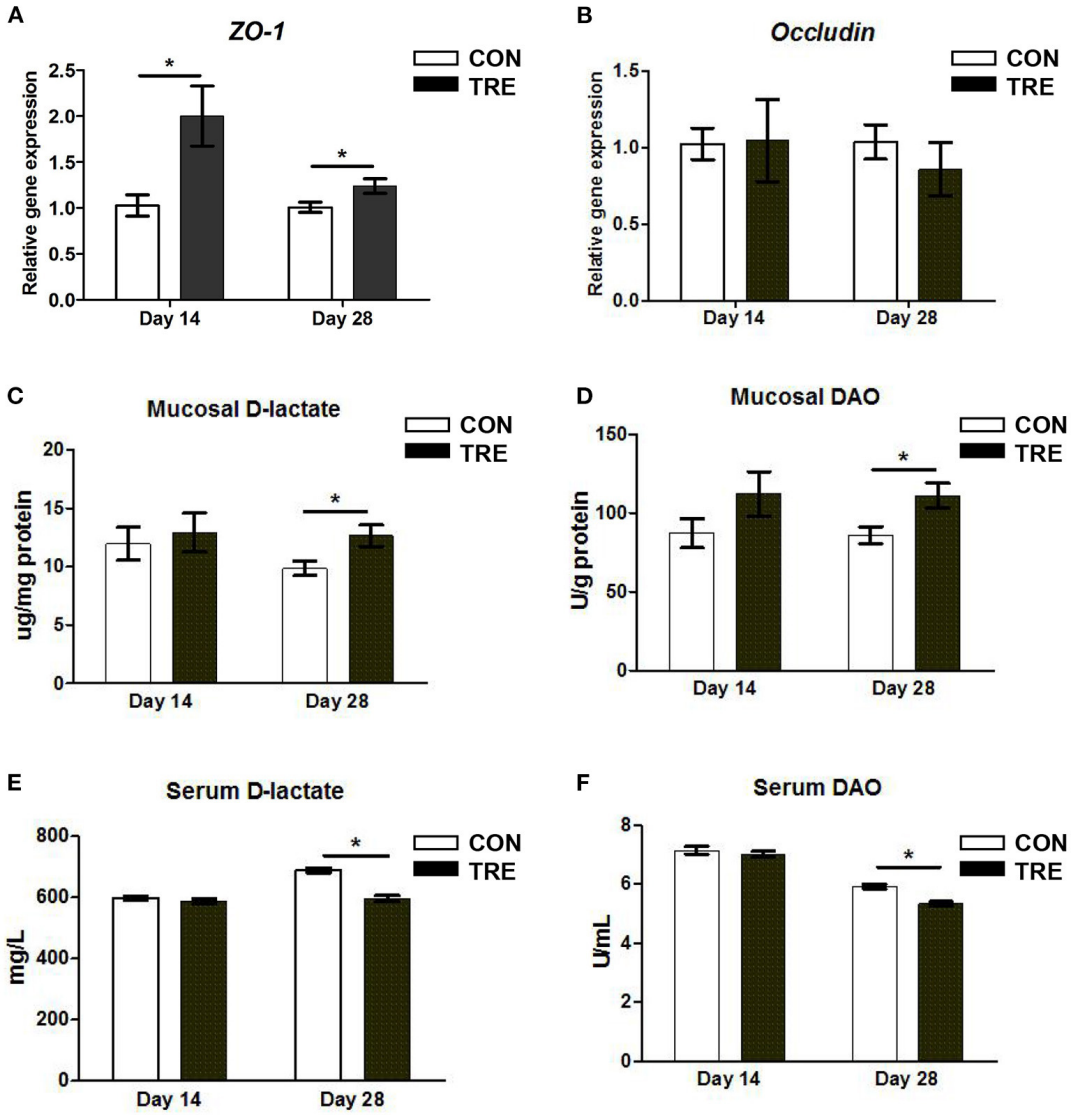
Effect of protein restriction and succedent realimentation on the barrier function of jejunum in weaned piglets (*n* = 6). **(A)** Alterations of *zonula occludens 1* (*ZO-1)* genes expression in jejunal mucosa on day 14 and 28. and **(B)** Alterations of *occludin* genes expression in jejunal mucosa on day 14 and 28. **(C)** Alterations of D-lactate levels in jejunal mucosa on day 14 and 28. **(D)** Alterations of diamine oxidase (DAO) level in jejunal mucosa on day 14 and 28. **(E)** Alterations of D-lactate level in serum. **(F)** Alterations of diamine oxidase (DAO) level in serum. *P* < 0.05 were considered significant and markers (^*^) between groups on the same day. CON, control group; TRE, treatment group.

The changed IGF-1 content was closely associated with gut development and function especially in maintaining barrier function. Thus, we measured the gene expression level of occludin and zonula occludens 1 (ZO-1) in jejunal mucosa. As shown in Figures 2A,B, in response to protein restriction and succedent realimentation, the *ZO-1* gene expression level was up-regulated in jejunal mucosa of TRE piglets on day 14 and day 28 (*p* < 0.05). However, protein restriction and succedent realimentation did not affect the alteration of occludin gene expression level in the CON and TRE groups (*p* > 0.05). To further reveal the relationship between the gene expression of barrier protein and gut permeability, we measured the D-lactate content and DAO activity in jejunal mucosa and serum. It is well-accepted that gut bacteria-derived D-lactate and DAO produced by epithelial cells were endogenous markers to reflect gut permeability. When the intestinal mechanical barrier is damaged, D-lactate and DAO would be released into the blood. As shown in Figures 2C–F, protein restriction did not change the D-lactate content and DAO activity in jejunal mucosa and serum in the CON and TRE groups on day 14. However, after the protein succedent realimentation, the D-lactate content and DAO activity in jejunal mucosa of TRE piglets were higher than those of CON piglets on day 28 (*p* < 0.05). In addition, compared with the CON piglets, TRE piglets had significantly decreased the serum D-lactate content and DAO activity on day 28 (*p* < 0.05).

### Effect of Protein Restriction and Succedent Realimentation on Nitrogen Nutrient Digestibility and Transporter Genes Expression of Jejunum in Weaned Piglets

**Figure 3:**
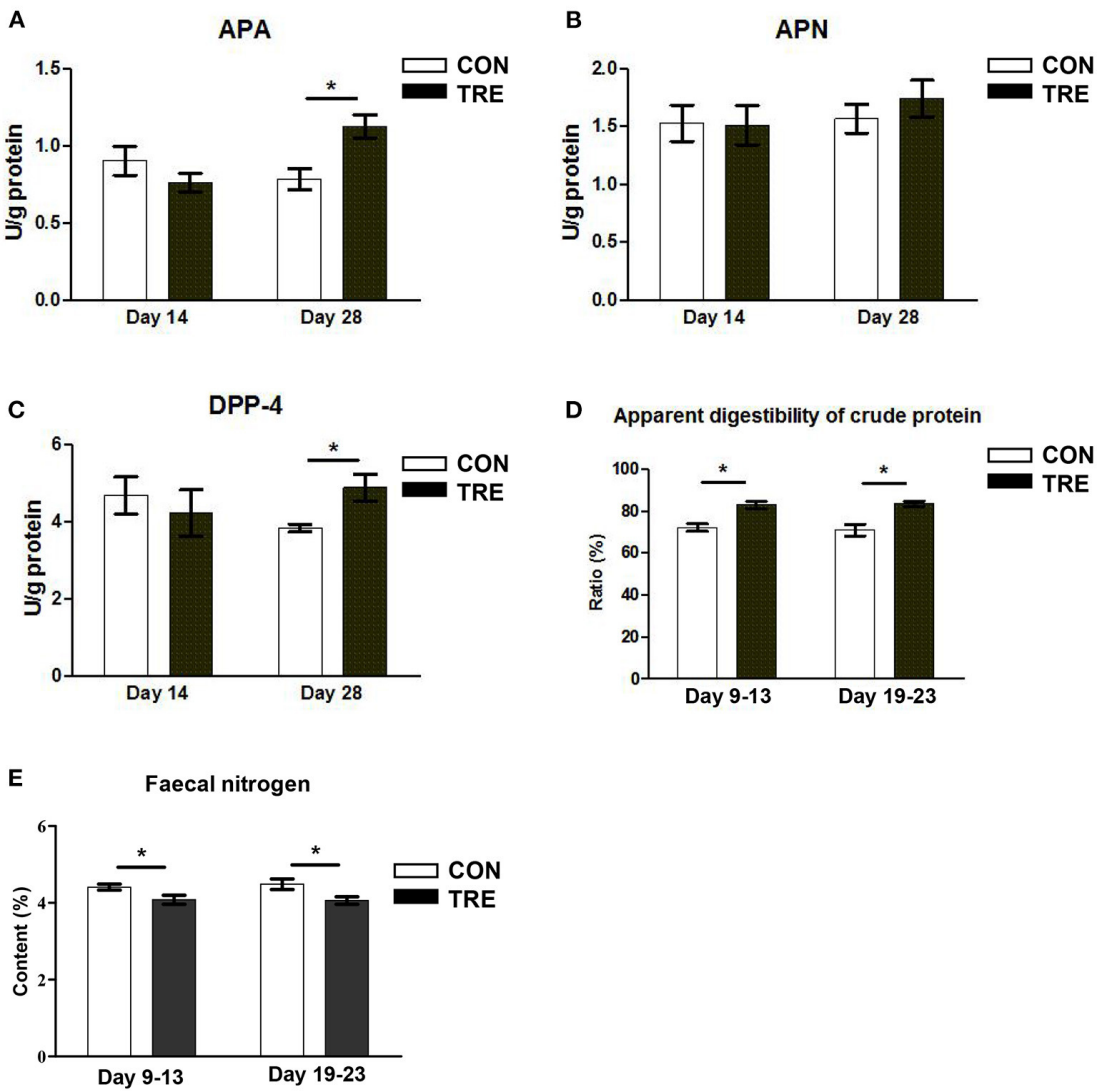
Effect of protein restriction and succedent realimentation on nitrogen nutrient digestibility of jejunum in weaned piglets (*n* = 6). **(A)** Alterations of aminopeptidases A (APA) level in jejunal mucosa on day 14 and 28. **(B)** Alterations of aminopeptidases N (APN) level in jejunal mocusa on day 14 and 28. **(C)** Alterations of dipeptidyl peptidase-4 (DPP-4) level in jejunal mucosa on day 14 and 28. **(D)** and **(E)** Alterations of fecal apparent digestibility of crude protein and nitrogen content on day 14 and 28. *P* < 0.05 were considered significant and markers (^*^) between groups on the same day. CON, control group; TRE, treatment group.

To investigate whether the protein restriction and succedent realimentation would regulate the nitrogen nutrient digestibility of jejunum in weaned piglets, we first measured the enzyme activity of several peptidases. As shown in Figures 3A–C, the enzyme activities of APA and DPP-4 were not significantly changed in the CON and TRE group on day 14 (*p* > 0.05). On day 28, the protein succedent realimentation increased the enzyme activities of APA and DPP-4 in TRE piglets jejunal mucosa (*p* <0.05). In addition, neither protein restriction nor succedent realimentation affected the jejunal mucosal enzyme activity of APN on days 14 and 28 (*P* > 0.05). We also investigated how protein restriction and succedent realimentation regulated the gene expression of transporters including glucose transporters 2 (Glut2), sodium/glucose cotransporters 1 (Sglt1), and peptide transporters 1 (Pept-1) in jejunal mucosa of piglets. As shown in Table 1, the protein restriction and succedent realimentation decreased the gene expression of *Glut2* and increased the gene expression of *Sglt1* in the colonic mucosa of TRE piglets on days 14 and 28. Both protein restriction and protein succedent realimentation significantly up-regulated the gene expression of *Pept-1* in jejunal mucosa of TRE piglets on days 14 and 28 (*p* < 0.05). To further investigate the nitrogen nutrient digestibility of jejunum in weaned piglets, we measured the apparent digestibility of crude protein and nitrogen content in the feces of piglets. As shown in Figures 3D,E, the fecal apparent digestibility of crude protein was significantly increased, while the fecal nitrogen content was significantly decreased in TRE piglets during the protein restriction and succedent realimentation (*p* < 0.05).

**Table 1:** Effect of protein restriction and succedent realimentation on transporter genes expression of jejunum in weaned piglets (*n* = 6).

**Item**	**CON**	**TRE**	**SEM**	***p-Value***
**Day 14**				
*Glut2*	1.01	0.55*	0.07	0.003
*Sglt1*	1.00	0.98	0.11	0.914
*PepT-1*	1.00	1.64*	0.10	0.005
**Day 28**				
*Glut2*	1.13	1.12	0.26	0.980
*Sglt1*	1.03	1.68*	0.14	0.014
*PepT-1*	1.00	1.29*	0.07	0.045

P < 0.05 were considered significant and markers (*) between groups on the same day. CON, control group; TRE, treatment group; SEM, standard error of mean; Glut2, glucose transporters 2; Sglt1, sodium/glucose co-transporters 1; Pept-1, peptide transporters 1.

### Effect of Protein Restriction and Succedent Realimentation on Bacterial Diversity of Different Colonic Niches in Weaned Piglets

**Figure 4:**
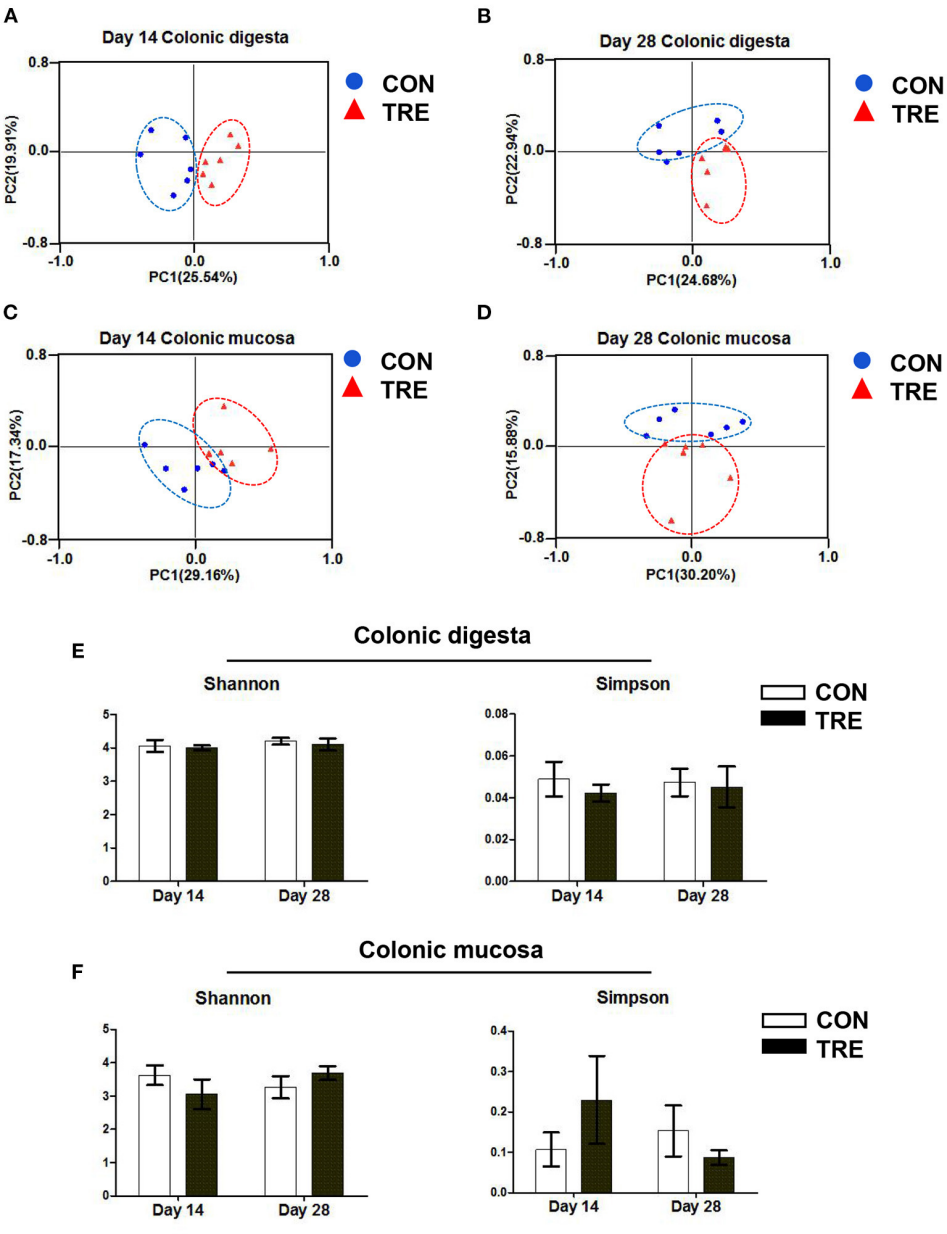
Effect of protein restriction and succedent realimentation on bacterial diversity of different colonic niches in weaned piglets (*n* = 6). **(A)** Alterations of β-diversity in colonic digesta on day 14. **(B)** Alterations of β-diversity in colonic digesta on day 28. **(C)** Alterations of β-diversity in colonic mucosa on day 14. **(D)** Alterations of β-diversity in colonic mucosa on day 28. **(E)** Alterations of α-diversity in jejunal digesta on day 14 and 28. **(F)** Alterations of α-diversity in jejunal mucosa on day 14 and 28. *P* < 0.05 were considered significant and markers (^*^) between groups on the same day. CON, control group; TRE, treatment group.

Several studies have demonstrated that lumen and mucosa had different gut bacterial compositions (19, 24). Therefore, to comprehensively investigate how the protein restriction and succedent realimentation influenced the colonic bacterial composition in weaned piglets, we analyzed the bacterial composition in different colonic niches (mucosa and digesta) of weaned piglets by using 16S rRNA MiSeq sequencing. As shown in Figures 4A–D, based on the principal co-ordinates analysis (PCoA) of β diversity, we observed that the plots representing either colonic digesta bacteria or colonic mucosal bacteria were markedly separated between two groups on day 14 and day 28 in response to the protein restriction and succedent realimentation. Additionally, we evaluated the stability of the colonic bacterial community in CON and TRE groups by calculating the α diversity indexes of Shannon and Simpson. As shown in Figures 4E,F, the protein restriction and succedent realimentation did not significantly affect the alteration of Shannon and Simpson between the CON and TRE piglets on day 14 and day 28 (*p* > 0.05).

### Effect of Protein Restriction and Succedent Realimentation on Bacterial Composition of Different Colonic Niches in Weaned Piglets

**Figure 5:**
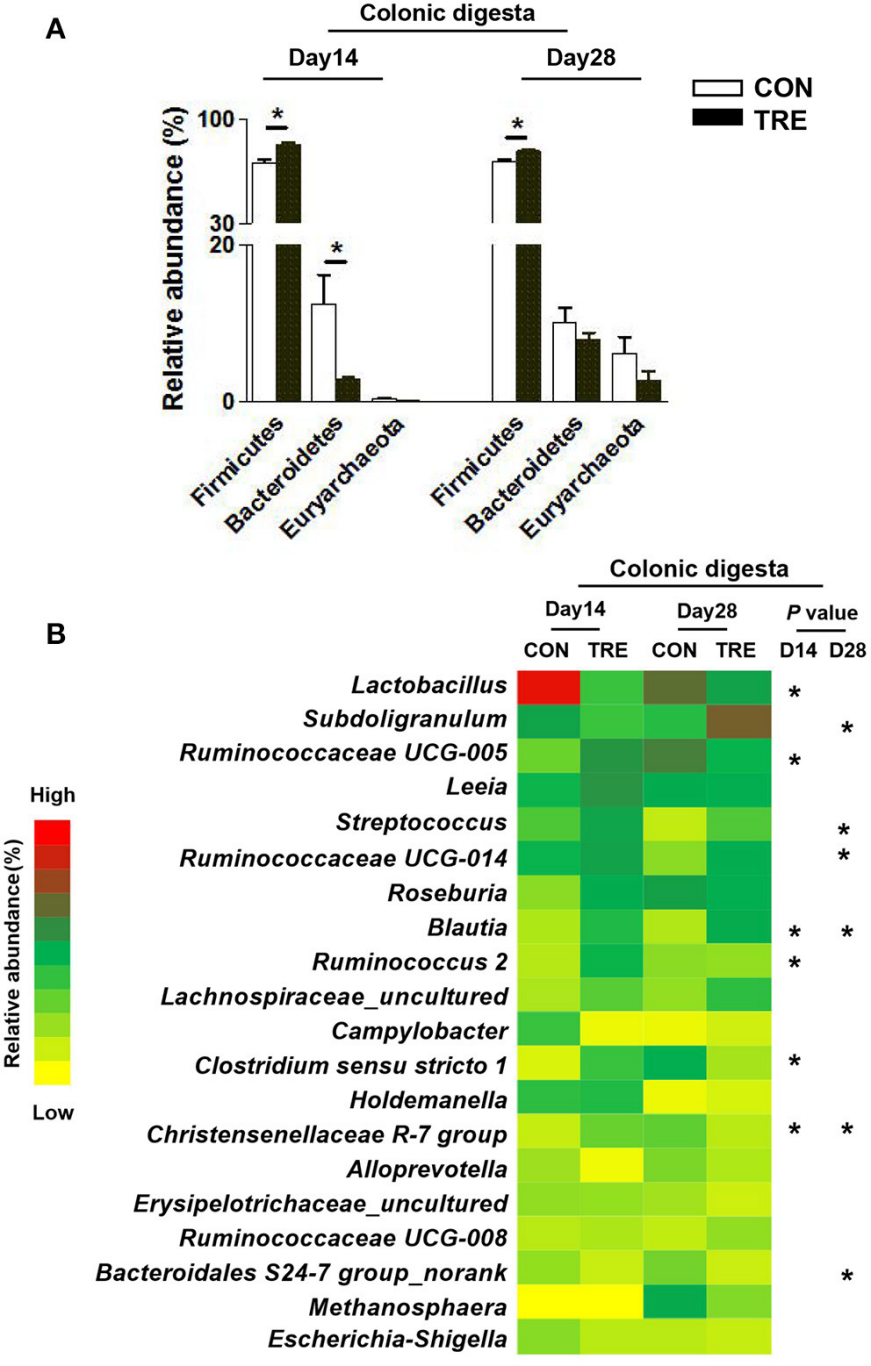
Effect of protein restriction and succedent realimentation on bacterial composition of colonic digesta in weaned piglets (*n* = 6). **(A)** Alterations of dominant phyla in colonic digesta on day 14 and 28. **(B)** Alterations of dominant genera in colonic digesta on day 14 and 28. *p* < 0.05 were considered significant and markers (*) between groups on the same day. CON, control group; TRE, treatment group.

**Figure 6:**
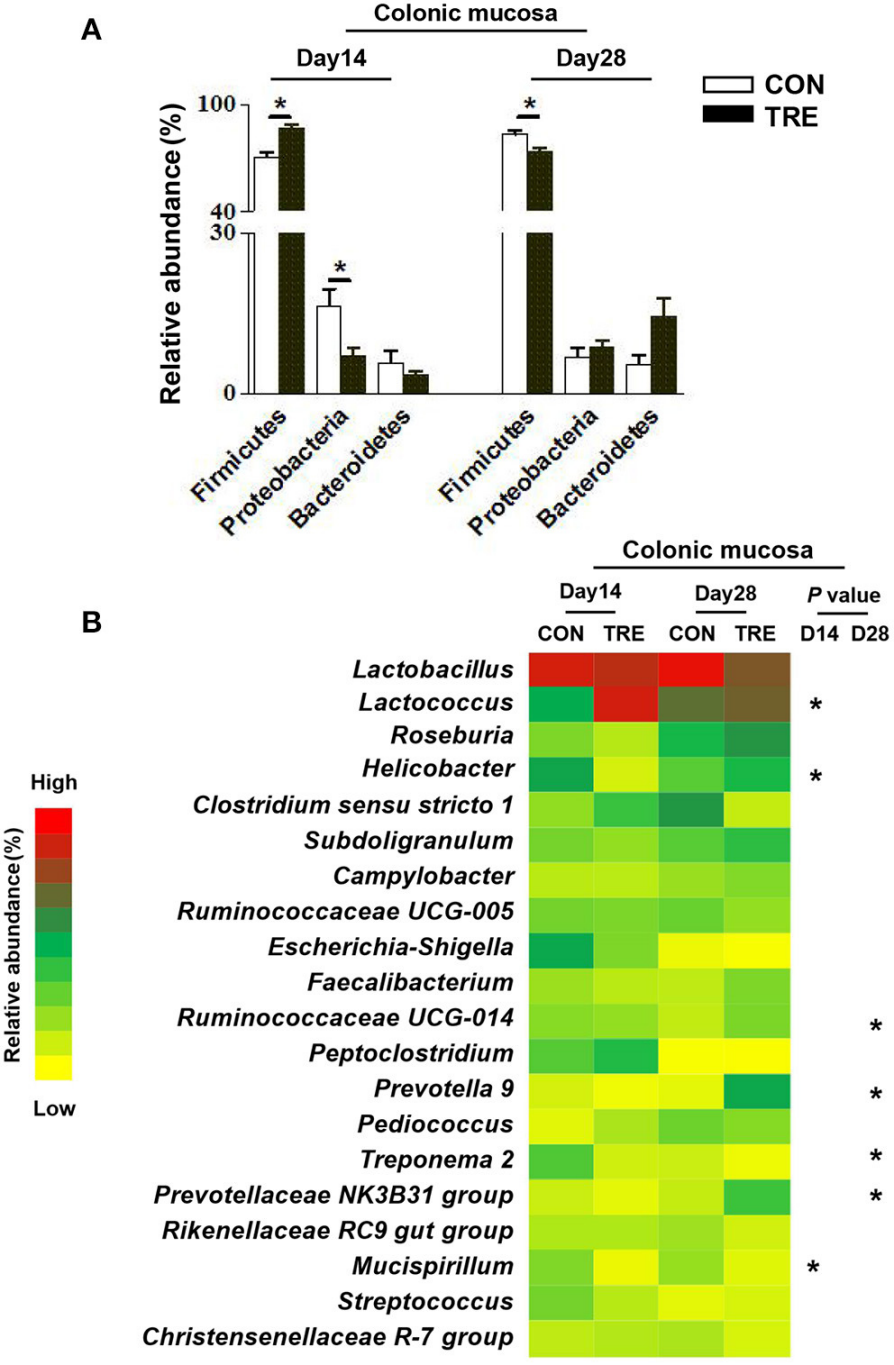
Effect of protein restriction and succedent realimentation on bacterial composition of colonic mucosa in weaned piglets (*n* = 6). **(A)** Alterations of dominant phyla in colonic mucosa on day 14 and 28. **(B)** Alterations of dominant genera in colonic mucosa on day 14 and 28. *p* < 0.05 were considered significant and markers (*) between groups on the same day. CON, control group; TRE, treatment group.

We investigated how protein restriction and succedent realimentation influenced the colonic bacterial composition on days 14 and 28. After categorizing the sequencing data, four dominant phyla (the total of their relative abundance over 90%) including the Firmicutes, Bacteroidetes, Proteobacteria, and Euryatchaeotawere were detected at the phylum level in different colonic niches (Figures 5A, 6A). As shown in Figure 5A, protein restriction increased the relative abundance of Firmicutes in colonic digesta, but decreased the relative abundance of Bacteroidetes on day 14 (*p* < 0.05). After the protein succedent realimentation, the relative abundance of Firmicutes was significantly increased in colonic digesta of TRE piglets on day 28 (*P* < 0.05). In addition, as shown in Figure 6A, the protein restriction increased the relative abundance of Firmicutes in the colonic mucosa, but decreased the relative abundance of Proteobacteria on day 14 (*p* < 0.05). Moreover, the protein succedent realimentation significantly decreased the relative abundance of Firmicutes in the colonic mucosa of TRE piglets on day 28 (*p* < 0.05).

Afterward, we selected the top 20 genera as dominant genera to statistically investigate the changes in their relative abundance in colonic mucosa and digesta (Figures 5B, 6B). The protein restriction significantly increased the relative abundance of *Ruminococcaceae UCG-005, Blautia, Ruminococcus 2, Clostridium sensu stricto 1*, and *Christensenellaceae R-7* group in colonic digesta on day 14, while deceased the relative abundance of *Lactobacillus* (*p* < 0.05; Figure 5B). Moreover, the protein succedent realimentation significantly increased the relative abundance of *Subdoligranulum, Streptococcus, Ruminococcaceae UCG-014*, and *Blautia* on day 28, while deceased the relative abundance of *Christensenellaceae R-7* group and *Bacteroidales S24-7 group_norank* (*P* < 0.05; Figure 5B). The protein restriction significantly increased the relative abundance of *Lactococcus* on day 14 in the colonic mucosa, while deceased the relative abundance of *Helicobacter* and *Mucispirillum* (*P* < 0.05; Figure 6B). In addition, the protein succedent realimentation significantly increased the relative abundance of *Ruminococcaceae UCG-014, Prevotella 9*, and *Prevotellaceae NK3B31 group* on day 28, while decreasing the relative abundance of *Treponema 2* (*p* < 0.05; Figure 6B).

### Effect of Protein Restriction and Succedent Realimentation on the Concentration of SCFAs and Ammonia-N in Colonic Digesta of Weaned Piglets

To reveal how the protein restriction and succedent realimentation influence the colonic bacterial metabolism in weaned piglets, we measured the concentration of SCFAs and ammonia-N in the colonic lumen of weaned piglets. As shown in Table 2, on day 14, the protein restriction decreased the concentration of isobutyrate, isovalerate, total branch chain fatty acids, and ammonia-N in the colonic lumen of TRE piglets (*p* < 0.05). On day 28, the protein succedent realimentation significantly increased the concentration of acetate, propionate, butyrate, and total SCFAs, while decreasing the concentration of isovalerate, total BCFAs, and ammonia-N in TRE piglets colonic lumen (*p* < 0.05).

**Table 2:** Effect of protein restriction and succedent realimentation on the concentration of SCFAs and ammonia-N in colonic digesta of weaned piglets (*n* = 6).

**Item**	**CON**	**TRE**	**SEM**	***P*** **-value**
**Day 14**				
Acetate (μmol/g digesta)	35.06	31.63	2.28	0.952
Propionate (μmol/g digesta)	12.90	11.01	0.78	0.402
Isobutyrate (μmol/g digesta)	0.25	0.14^*^	0.02	0.023
Butyrate (μmol/g digesta)	4.23	3.12	0.40	0.329
Isovalerate (μmol/g digesta)	0.33	0.16^*^	0.02	0.004
Valerate (μmol/g digesta)	0.79	0.52	0.08	0.092
Total SCFAs (μmol/g digesta)	53.55	46.58	3.12	0.646
BCFAs (μmol/g digesta)	0.58	0.30^*^	0.04	0.007
Ammonia-N (μg/g digesta)	234.48	121.57^*^	27.06	0.012
**Day 28**				
Acetate (μmol/g digesta)	42.70	55.83^*^	1.98	0.001
Propionate (μmol/g digesta)	12.74	19.27^*^	0.64	<0.001
Isobutyrate (μmol/g digesta)	0.22	0.20	0.05	0.842
Butyrate (μmol/g digesta)	5.35	7.99^*^	0.62	0.015
Isovalerate (μmol/g digesta)	0.41	0.24^*^	0.02	0.001
Valerate (μmol/g digesta)	0.82	0.79	0.09	0.817
Total SCFAs (μmol/g digesta)	62.24	84.32^*^	2.49	<0.001
BCFAs (μmol/g digesta)	0.63	0.44^*^	0.07	0.038
Ammonia-N (μg/g digesta)	206.37	128.24^*^	13.40	0.002

p < 0.05 were considered significant and markers (^*^) between groups on the same day. CON, control group; TRE, treatment group; SEM, standard error of mean; SCFAs, short chain fatty acids; BCFAs, branch chain fatty acids.

## Discussion

To obtain the health benefits of protein restriction without inhibiting the growth of weaning piglets, we attempted to use the strategy of protein restriction and succedent realimentation. The previous study of our group showed that a 2-week protein restriction for post-weaning piglets reduced their body weight. The following 2-week of the diet protein realimentated to normal level increased the bodyweight of protein restriction piglets similar to the weight of control piglets (10). Notably, the piglets which experienced the protein restriction and succedent realimentation exhibited a lower diarrhea rate than the control piglets which fed a diet with a normal protein level in the whole experimental period (10). Consistent with our group's results, Hou et al. had a similar finding in their study of protein restriction and subsequent realimentation on weaned piglets (4). These data suggested that protein restriction and succedent realimentation is an effective strategy for obtaining the health benefits of protein restriction without inhibiting the growth of weaning piglets. Indeed, the weaning piglets exhibited a typical compensatory growth (or catch-up) during the protein succedent realimentation (25). Compensatory growth was defined as a physiological process whereby domestic animals accelerate their growth after a period of growth retarded caused by feed restriction (26, 27). Hornick et al. highlighted that the mechanisms underlying compensatory growth were associated with the changes in several hormone secretions including GH, insulin, and IGF-1 (26). Basis of this view, the previous study of our group reported that the protein realimentation activated the hepatic GH-IGF1 axis of weaning piglets during the compensatory growth (11, 23). Moreover, Ju and co-workers confirmed that the growth inhibition caused by the early protein restriction could be compensated through compensatory growth in growing pigs, and the mechanism of compensation is related to regulating the expression level of GH, IGF-1, GH-R, and IGF-1-R (28). These findings implied that the GH-IGF1 axis plays a key role in compensatory growth piglets. Notably, as an important organ for the digestion and absorption of dietary nutrients, the intestine is one of the main target organs for IGF-1, and its function and development are closely related to the secretion of IGF-1. In this study, we found that the protein restriction did not affect the jejunal expression level of hormones and IGF-1R on day 14, while the protein succedent realimentation made the jejunal expression level of IGF-1 and IGF-1R in TRE piglets higher than that of CON piglets on day 28. A recent study described that IGF-1 and/or IGF-1R entered the cell nuclei through a nuclear pore complex, and the nuclear localization of IGF-1 and/or IGF-1R regulated the transcription of genes and increased the nuclear residence time of signaling molecules activated by IGF-1 (29). Thus, the increased expression level of IGF-1 and IGF-1R may modulate the jejunal function and make a contribution to the compensatory growth in TRE piglets during the protein succedent realimentation.

Maintaining small intestinal epithelial integrity is a critical event for the growth and health of weaning piglets. A review article pointed out that weaning piglets fed on a low protein diet would help to keep small intestinal barrier function by reducing the risk of infection caused by proteolytic microbes, such as *Escherichia coli* and *Clostridrium perfringens* (6). In line with this view, we found that the protein restriction increased the jejunal gene expression of barrier protein *ZO-1* on day 14. Interestingly, our results also showed that the protein succedent realimentation increased the jejunal gene expression of barrier protein *ZO-1* and decreased jejunal permeability in weaning piglets on day 28. Ko et al. revealed that IGF-1 up-regulated the tight-junction protein ZO-1 in A431 cells (30). Meanwhile, a previous study also confirmed that IGF-1 could reduce intestinal permeability in rats with a bile duct ligation, and enhanced the barrier function of the intestinal epithelial cell monolayers where lipopolysaccharide caused a decrease of transepithelial electrical resistance that was reversed by IGF-1 (31). Thus, consistent with the changes of jejunal IGF-1 content mentioned above, the protein succedent realimentation increased the content of IGF-1, which may improve the jejunal integrity of piglets on day 28. Notably, the small intestinal epithelial integrity in the piglets is a fundamental basis of small intestinal digestion and absorption. Small intestinal epithelial cells show a polar structure with an apical membrane facing the lumen, while the apical membrane of the small intestine harbors different peptidases and transporters for nutrient digestion and absorption (32). In this study, the protein restriction increased the gene expression level of *Pept1* in the jejunum of piglets on day 14. In addition, the protein succedent realimentation not only increased the *Pept-1* gene expression level but also enhanced the gene expression level of *Sglt1* and enzyme activities of APA and Dpp-4 in piglets jejunum on day 28. Odenwald et al. highlighted the multiple ZO-1 mediated interactions contributed to the coordination of epithelial actomyosin function and the genesis of unified apical surfaces (33). Here, we logically postulated that the increased jejunal epithelial integrity could improve the gene expression of transporters and the activities of peptidases in jejunal apical membrane of piglets on days 14 and 28. In addition, consistent with the alteration of the *Pept-1* gene expression level and peptidases activity, we found that the protein restriction and succedent realimentation decreased the apparent digestibility of crude protein and nitrogen content in the feces of piglets. All these data suggested that the protein restriction and succedent realimentation promoted jejunal nitrogen nutrient digestibility through maintaining epithelial integrity.

Colon, as the main segment for gut bacterial colonization, harbors a huge number of bacteria. Moreover, the colonic bacteria colonized in different niches (mucosa and lumen) of piglets often showed different compositions and functions (24). To comprehensively understand the effects of protein restriction and succedent realimentation on colonic microbial composition in weaning piglets, we investigated the microbial composition in colonic mucosa and lumen. Previous studies have confirmed that different dietary protein levels could modulate the colonic bacterial composition of piglets (34, 35). Based on patterns of Pcoa, we found that both the protein restriction and the protein succedent realimentation changed the bacterial composition of different colonic niches on day 14 and 28. In agreement with this finding, the protein restriction and succedent realimentation increased the ratio of Firmicutes to Bacteroidetes at the phylum level in colonic digesta on days 14 and 28. Similar findings were also confirmed by Hou et al., whose results suggested that the increased ratio of Firmicutes to Bacteroidetes in colonic digesta was closely related to the body fat deposition during the protein restriction and succedent realimentation (4). In addition, the protein restriction and succedent realimentation also changed the relative abundances of dominant genera in different colonic niches on days 14 and 28. We found that the protein restriction sharply decreased the relative abundance of *Lactobacillus* in the colonic digesta of piglets on day 14. Although *Lactobacillus* is known to be probiotics, the relative abundance of *Lactobacillus* in the colon weaning piglets does no longer absolutely dominate like that in the foregut (36). Meanwhile, Gresse et al. reported that several important commensal bacteria such as *Ruminococcus 2, Ruminococcaceae UCG-005, Ruminococcaceae UCG-014*, and *Subdoligranulum* gradually become the core microbiota in the piglets hindgut from the distal ileum (36). Consistent with their findings, we found that the protein restriction and succedent realimentation increased the relative abundance of *Ruminococcaceae UCG-005, Ruminococcus 2, Subdoligranulum*, and *Ruminococcaceae UCG-014* in the colonic digesta of TRE piglets on day 14 and 28. Interestingly, both the protein restriction and the protein succedent realimentation markedly increased the relative abundance of *Blautia* in the colonic digesta of weaning piglets on days 14 and 28. Previous studies pointed out that *Blautia* exhibits potential probiotic properties through relieving gut inflammatory responses (37, 38). Our data showed that the protein restriction and succedent realimentation promoted the colonization of commensal and beneficial bacteria like *Blautia* in the colonic digesta of weaning piglets, which would help to maintain the colonic ecosystem homeostasis. Mucosa-associated bacteria were like a “carpet” covering the colonic mucosa, and participated in the formation of biological barrier. These mucosal bacteria directly modulated mucosal immune response via binding pattern recognition receptors or secreting antimicrobial-like agents (18). In this study, the protein restriction increased the relative abundance of *Lactococcus* and the protein succedent realimentation increased the relative abundance of *Ruminococcaceae UCG-014, Prevotella 9*, and *Prevotellaceae NK3B31 group* in the colonic mucosa of TRE piglets on day 14 and day 28. The bacteria that belonged to the *Prevotella* genus are one of the most predominant genera among the intestinal bacteria in both pre-and post-weaned piglets. They are attracting more interest as a commensal that is present in the hindgut because of their ability to degrade diet fiber and oligosaccharides which could modulate the host mucosal immune development (18, 39). Moreover, the members of *Ruminococcaceae* are defined as important commensal bacteria in the piglet intestine which could help to maintain gut immune homeostasis (18). Of note, the protein restriction and succedent realimentation decreased the relative abundance of *Helicobacter, Mucispirillum* and *Treponema 2* in the colonic mucosa of TRE piglets on day 14 and 28. Previous studies showed that some species that belong to *Helicobacter, Mucispirillum* and *Treponema 2* triggered infection and inflammatory response in the gastrointestinal tract mucosa (40, 41). Thus, these results implied that the protein restriction and succedent realimentation reduced the colonization of potential pathogens in the colonic mucosa weaning piglets, which would help to maintain the colonic health by modulating colonic immune homeostasis.

Besides altering the composition of colonic bacteria, the diet protein level influenced the concentration of colonic bacterial metabolites in weaning piglets which affects gut health and host metabolism. Ammonia is one of the major products of the dietary protein fermentation performed by intestinal bacteria, and excessive ammonia production would damage gut health (42). Yokoo et al. found that ammonia impaired the tight junction barrier by increasing oxidative stress and impairing mitochondrial function in Caco-2 cells (43). In the present study, our data showed that the protein restriction and succedent realimentation decreased the ammonia-N concentration in the colon of TRE piglets on days 14 and 28. We also found that the protein restriction and succedent realimentation decreased the concentrations of BCFAs which were other products of colonic microbial fermentation on dietary protein (42). Thus, our data suggested that the protein restriction and succedent realimentation could decrease the dietary fermentation by colonic bacteria and help to maintain colon health. Interestingly, the protein succedent realimentation dramatically increased the concentrations of SCFAs in TRE piglets colon on day 28. Our results indicated that the protein succedent realimentation could increase the abundances of SCFAs producers in different colonic niches of TRE piglets on day 28. For example, the *Ruminococcaceae UCG-014, Ruminococcaceae UCG-014, Prevotella 9*, and *Prevotellaceae NK3B31 group* are acetate producers, while the *Subdoligranulum* and *Blautia* are classical butyrate producers (44, 45). The previous studies have revealed that the SCFAs produced by gut microbiota improved the gut barrier function and modulated gut immune homeostasis by the activation of the adenosine 5‘-monophosphate-activated protein kinase pathway and the inhibition of the nuclear factor kappa-B pathway (18, 46). In addition, most SCFAs exist in the form of ions in the colon, and they are absorbed by ion transporters located in epithelial cells of the colon (47). When SCFA ions are absorbed, they are the main energy source for colonic epithelial cells. Zhou et al. illustrated that the weaning process has a significant effect on colonic growth and development, which is associated with the change of SCFAs concentrations in the colon (48). Thus, our results indicated that the increased SCFAs production might be effective to maintain colonic physiology function and improve colonic growth. Importantly, the previous study confirmed that SCFAs produced when microbiota ferment fiber and induce hormone IGF-1 which promotes bone growth and remodeling (13). Moreover, Xia et al. also found that coix seed polysaccharides can increase the levels of SCFAs through the gut microbiota, thereby activating the signaling pathways downstream of IGF-1 and generating hypoglycemic effects (49). In summary, these findings suggested that the protein succedent realimentation increased the SCFAs production by colonic bacteria, and made a contribution to compensatory growth of weaning piglets via promoting IGF-1 secretion on day 28.

## Conclusion

The protein restriction and succedent realimentation increased the barrier function and nitrogen nutrient digestibility in weaning piglets jejunum. The process also promoted the relative abundances of beneficial bacteria and decreased the potential pathogens colonization in different colonic niches of weaning piglets. All these findings demonstrated that the strategy of protein restriction and succedent realimentation is an effective way to improve the intestinal health of weaning piglets.

## Data Availability Statement

The datasets presented in this study can be found in online repositories. The names of the repository/repositories and accession number(s) can be found in the article/Supplementary Material.

## Ethics Statement

The animal study was reviewed and approved by the Animal Care and Use Committee of Nanjing Agricultural University (Nanjing, Jiangsu province, China). Written informed consent was obtained from the owners for the participation of their animals in this study.

## Author Contributions

JW, YZ, and ST contributed to conception and design of the study. QS organized the database. YZ and HY performed the statistical analysis. JuW wrote the first draft of the manuscript. JiW wrote sections of the manuscript. JiW and WZ edited the manuscript. All authors contributed to manuscript revision, read, and approved the submitted version.

## Funding

This study was supported by the National Key R&D Program of China (2017YFE0135200).

## Conflict of Interest

The authors declare that the research was conducted in the absence of any commercial or financial relationships that could be construed as a potential conflict of interest.

## Publisher's Note

All claims expressed in this article are solely those of the authors and do not necessarily represent those of their affiliated organizations, or those of the publisher, the editors and the reviewers. Any product that may be evaluated in this article, or claim that may be made by its manufacturer, is not guaranteed or endorsed by the publisher.
