# Supplementary material for: Effects of Protein Restriction and Succedent Realimentation on Jejunal Function and Bacterial Composition of Different Colonic Niches in Weaned Piglets
Source: Front Vet Sci. 2022 May 3;9:877130. doi: 10.3389/fvets.2022.877130 (PMC9111176; doi:10.3389/fvets.2022.877130)
Supplement: Supplementary file 1 [file Data_Sheet_1.docx]

**Table S1** The ingredient and nutrient composition of different diets for weaning piglets.

| **Item** | **NPD** | **LPD** |
| --- | --- | --- |
|  |  |  |
| **Ingredients (kg/100kg)** |  |  |
| Corn | 41.62 | 60.91 |
| Cornstarch | 18.30 | 16.22 |
| Soybean meal | 25.97 | 11.20 |
| Fishmeal | 5.00 | 1.41 |
| Soybean oil | 4.00 | 4.00 |
| L-lysine | 0.70 | 1.30 |
| DL-methionine | 0.23 | 0.34 |
| L-threonine | 0.18 | 0.50 |
| L-tryptophan | 0.01 | 0.12 |
| Premixa | 4.00 | 4.00 |
| Total | 100.00 | 100.00 |
| **Calculated nutrient levels** |  |  |
| Digestible energy, Mcal/kg | 3.49 | 3.54 |
| Crude protein, % | 18.83 | 13.05 |
| Lysine, % | 1.52 | 1.51 |
| Methionine + Cysteine, % | 0.83 | 0.74 |
| Threonine,% | 0.91 | 0.92 |
| Methionine,% | 0.55 | 0.54 |
| Dry matter,% | 90.26 | 89.83 |
| Tryptophan,% | 0.25 | 0.25 |

NPD, normal protein diet; LPD, low protein diet.

^a^ The premix per kg contains vitamin A (KIU) 100-400, vitamin D3 (KIU) 25-215, vitamin E (mg) ≥ 300, vitamin K3 (mg) ≥ 30, vitamin B1 (mg) ≥ 25, vitamin B2 ≥ 75, vitamin B6 ≥ 35, vitamin B12 (mg) ≥ 0.25, niacin (mg) ≥ 400, pantothenic acid (mg) ≥ 300, folic acid (mg) ≥ 14, D-biotin (mg) ≥ 1.0, choline chloride (mg) ≥ 5,000, Cu (mg) 500-5,000, Fe (mg) 500-7,500, Zn (mg) 500-3,750, Mn (mg) 500- 3,750, I (mg) ≥ 3.0, Se (mg) 2.5-12.5, Ca (%) 10-20, total P (%) ≥ 1.5, sodium chloride (%) 5-14, arginine (%) ≥ 3.7, phytase (U) ≥ 20,000.

**Table S2** The primer sequences of colonic mucosal genes

| **Gene** | **Primer sequence (5’-3’)** | **Accession number** |
| --- | --- | --- |
|  |  |  |
| *β-actin* | F- ATGCTTCTAGACGGACTGCG | XM_003357928.4 |
|  | R- GTTTCAGGAGGCTGGCATGA |  |
| *Glut2* | F- CCTGCTTGGTCTATCTGCTGTG | NM_001097417.1 |
|  | R- TTGATGCTTCTTCCCTTTCTTT |  |
| *Sglt1* | F- CCACTTTCCCTATAAAACCTCAC | NM_001164021.1 |
|  | R-CTCCATCAAACTTCCATCCTCAG |  |
| *Pept-1* | F- GATGAAATGTGAGCGTATGGG | NM_214347.1 |
|  | R-AAAGAGGGAGGATCTGGAAAA |  |
| *Occludin* | F- ATGCTTTCTCAGCCAGCGTA | NM_001163647.2 |
|  | R- AAG GTTCCATAGCCTCGGTC |  |
| *ZO-1* | F- GAGGATGGTCACACCGTGGT | XM_021098896.1 |
|  | R- GGAGGATGCTGTTGTCTCGG |  |
| *IGF-1R* | F- GGGATGACGAGAGACATCTATGAG | XM_021082920.1 |
|  | R- GAAGGACCAGACTCAGAGTGC |  |

**Figure S1**


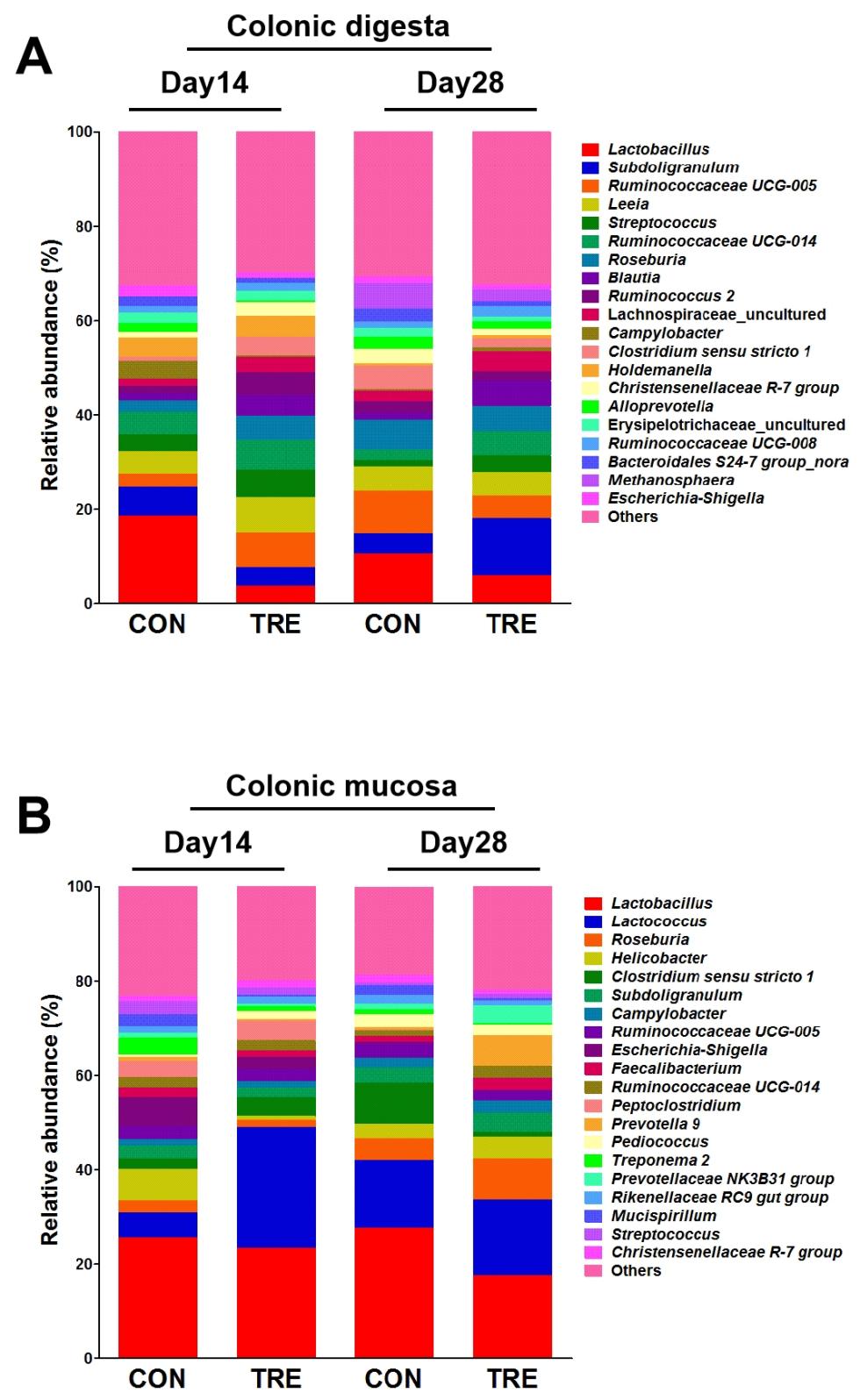


**Figure S1** Effect of protein restriction and succedent realimentation on dominant genus of colonic digesta (A) and mucosa (B) in weaned piglets (n = 6). CON, control group; TRE, treatment group.
